# Investigation of the Mechanism of Shengmai Injection on Sepsis by Network Pharmacology Approaches

**DOI:** 10.1155/2020/4956329

**Published:** 2020-08-03

**Authors:** Juan Lu, Xinkai Lyu, Ruiping Chai, Yue Yu, Minghui Deng, Xia Zhan, Zhengqi Dong, Xi Chen

**Affiliations:** ^1^Institute of Medicinal Plant Development, Chinese Academy of Medical Sciences, Peking Union Medical College, Beijing 100094, China; ^2^Key Laboratory of Cleaner Production and Integrated Resource Utilization of China National Light Industry, Beijing Technology and Business University, Beijing 100048, China

## Abstract

Shengmai injection (SMI) contains Ginsen Radix et Rhizoma Rubra, *Ophiopogon japonicus*, and Schisandrae Chinensis Fructus. It is used as a supportive herbal medicine in the management of sepsis, systemic inflammatory response syndrome, and septic or hemorrhagic shock. An UPLC method was established to identify and evaluate SMI fingerprints. Fingerprint similarities of 9 batches of SMI were compared. The network platform, “TCM-components-core targets-key pathways,” was established, and the mechanism of SMI in the treatment of sepsis was investigated. The similarity of 9 batches of SMI fingerprints was greater than 0.91. 44 peaks were selected as the common peaks, of which 11 peaks were identified. KEGG functional pathway analysis showed SMI was mainly involved in the pathways of cancer, cell cycle, and p53 signaling, suggesting SMI protects multiple organs via regulating immunity, inflammation, apoptosis, and energy metabolism. GO enrichment analysis showed active SMI components regulated various biological processes and altered the pathophysiology of sepsis. The interplays between SMI and multiple energy metabolism signaling cascades confer protection from life-threatening multiple organ failure in sepsis.

## 1. Introduction

Sepsis is a deregulated body response to infection, triggering inflammatory reactions that can cause systemic symptoms and damage multiple organs. Release of cytokines mediates uncontrolled inflammatory cascades that result in dysfunction and failure of multiple major organs and septic shock [1, 2]. Managing infection is the most critical strategy for sepsis therapy. However, these treatments could cause various side effects [3]. Clinically, sepsis is managed with early use of antibiotics and glucocorticoids. Traditional Chinese medicine, including Xuebijing injection [2, 4], Shenfu injection [5], and SMI [6], provides supportive effects in sepsis treatment.

Shengmai injection (SMI) origins from the ancient prescription of Chinese medicine Shengmaiyin; it contains Ginsen Radix et Rhizoma Rubra, *Ophiopogon japonicus*, and Schisandrae Chinensis Fructus. It invigorates Qi, nourishes Yin, and promotes blood circulation. SMI is used as add-on for supportive treatment in managing patients with sepsis, systemic inflammatory syndrome, and septic or hemorrhagic shock [7–9]. The frequency of adverse events associated with SMI is low [10, 11]. There have been few reports on the evaluation of SMI effective components and their underlying mechanisms despite they are used as supportive interventions for sepsis treatment. It is a compound with multiple components targeting multiple molecular networks; exploring its complex antisepsis mechanism in a suitable model is of great importance for sepsis treatment [12]. Network pharmacology is a useful tool for systemic investigation of the mechanisms of multiple component drugs [13, 14]. Its approach has been used to study “compound-protein/gene-disease” pathways which reveal complexities among drugs, biological systems, and diseases from a network perspective. Network pharmacology provides insights into the complex interrelationships between the active ingredients of traditional Chinese compounds and molecular mechanisms [15].

We established a fingerprint method to detect and represent chemical information of SMI. We mapped potential targets of SMI bioactive ingredients that may regulate the progress of sepsis using a network pharmacology approach. Our findings shed light on further understanding of the mechanisms of SMI in treating complex diseases such as sepsis.

## 2. Materials and Methods

### 2.1. Equipment and Reagents

Water Acquity H-class UPLC, equipped with quaternion high-pressure pump, automatic sampler, and PDA detector (Milford, USA); METTLER AB265-S electronic analytical balance (Zurich, Switzerland); and SB25-12DT ultrasonic cleaner (Ningbo, China) were used. Standard ginsenoside Rb1 (no. 171018), ginsenoside Rb2 (no. 171009), ginsenoside Rd (no. 170530), ginsenoside Re (no. 170924), ginsenoside RF (no. 171126), ginsenoside Rg1 (no. 180105), Ophiopogon D (no. 171126), schisandrol A (no. 180109), and schisandrin A (no. 171231) were all purchased from Shanghai Ronghe Medical Pharmaceutical Technology Co., Ltd (Shanghai, China). Ginsenoside Rb3 (no. 111686-201504) and ginsenoside Rg2 (no. 111779-200801) were purchased from the National Institutes for Food and Drug Control (Beijing, China). The purity of all of the above standards was above 98.0%. Acetonitrile and methanol were purchased from Merck (chromatographically pure, Darmstadt, Germany). Distilled water was purchased from Watson (Hong Kong, China). There were 9 batches of SMI: S1, 16120401005; S2, 160502; S3, 17071014; S4, 17040423; S5, 1704252; S6, 17091302; S7, 17092903; S8, 17061103; and S9, 17053005.

### 2.2. Standards and Sample Solution Preparation

Standard stock solutions of ginsenoside Rb1 (9.49 mg), ginsenoside Rb2 (10.22 mg), ginsenoside Rb3 (6.69 mg), ginsenoside Rd (6.24 mg), ginsenoside Re (6.24 mg), ginsenoside Rf (7.35 mg), ginsenoside Rg1 (13.24 mg), ginsenoside Rg2 (6.58 mg), Ophiopogon D (5.12 mg), schisandrol A (5.60 mg), and schisantherin A (3.27 mg) were dissolved in 5 ml methanol followed by sonication, respectively. Mixture of standard solution was filtered through the 0.22 *μ*m membrane in a 5 mL volumetric flask. Final concentration of each standard in the mixture was 37.96, 40.88, 26.76, 24.96, 24.96, 29.40, 52.96, 26.32, 20.48, 17.92, and 10.46 *μ*g·mL^−1^, respectively. SMI solution of each batch was filtered through the 0.22 *μ*m membrane before analysis.

### 2.3. UPLC Conditions

The analyte was separated by a Waters Acquity UPLC BEH C18 (2.1 mm × 50 mm, 1.7 *μ*m) column. The mobile phases used were solvent *A* (acetonitrile) and solvent *B* (water) with gradient elution (Table 1). The analysis was carried out at a flow rate of 0.3 mL/min. The column temperature was set to 40°C. UV detection wavelength was over the range of 190 to 400 nm. 5 *μ*L of the sample was injected. 210 nm was selected as the extraction wavelength of the fingerprints.

### 2.4. Precision of the Method

Method precision was determined by analyzing the same sample SMI (S1, 16120101005) five consecutive times in a day. The peak of schisantherin A was used as the reference peak. Relative standard deviation (RSD) was calculated from the relative peak area (RPA) or relative retention time (RRT) of each characteristic peak.

### 2.5. Sample Stability

Sample stability was evaluated using the same SMI (S1, 16120401005) after 0, 2, 4, 6, 8, 12, and 24 hours. The peak of schisantherin A was used as the reference peak. RSD was calculated from RPA or RRT of each peak to the reference peak from the chromatographic profiles of samples.

### 2.6. Repeatability

Repeatability was evaluated by analyzing six independently prepared SMI samples. The peak of schisantherin A was used as the reference peak. RSD was calculated from RPA or RRT of each peak to the reference peak from the chromatographic profiles of samples.

### 2.7. Information about Databases and Software of Network Pharmacology

TCMSP database (https://tcmspw.com/tcmsp.php), PubChem CID (https://pubchem.ncbi.nlm.nih.gov/search/), STITCH (http://stitch.embl.de/), Human Phenotype Ontology (HPO, https://hpo.jax.org/app/), STRING database (https://string-db.org), OMIM database (https://omim.org/), and DAVID database (https://david.ncifcrf.gov/summary.jsp) were used. Cytoscape software v3.5.1 was used.

### 2.8. Network Construction

Data acquisition and processing were done in databases which include SciFinder and TCMSP. Additionally, PubChem CID for each active ingredient of SMI was obtained from PubChem. We used SMILES format in STITCH chemical association networks and obtained the interaction complex between SMI bioactive components and the potential target protein in humans. Using HPO as a tool, we annotated and analyzed the core protein targets that participate in sepsis. The primary as well as predicted interactions between SMI target proteins and proteins involved in sepsis were analyzed in the STRING database. We collected core proteins that are highly associated with sepsis, while proteins with low correlation were filtered out [16]. The molecular interplays between SMI key targets and sepsis proteins were visualized in the Cytoscape platform. We calculated the degree, betweenness, and closeness of the targets; proteins with topological parameters greater than the corresponding median values were considered as major hits. The selected proteins were validated in the OMIM database to establish protein-disease association and construct “SMI Targets-Sepsis Targets” network.

### 2.9. Prediction of the SMI-Antisepsis Mechanism

A list of the selected top 20 key proteins was uploaded to the DAVID database for functional annotation and enrichment analysis to obtain the main pathways and network distribution that confer potential mechanisms for SMI treatment. Only pathways with *p* < 0.05 were considered for mechanism prediction.

## 3. Results and Discussion

### 3.1. Establishment of SMI Fingerprints and the Results of Methodological Evaluation

**Figure 1:**
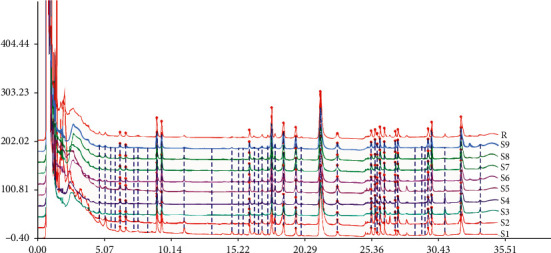
UPLC fingerprints of 9 batches of SMI.

**Figure 2:**
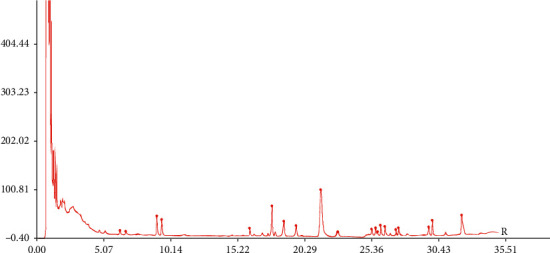
Contrast fingerprint of SMI.

The RSDs of RPA and RRT for precision, repeatability, and sample stability were lower than 3%, respectively. The results showed that the fingerprint method developed for analysis of SMI is reliable and applicable. Figures 1 and 2 show the UPLC chromatogram fingerprints of 9 batches of SMI.

### 3.2. Reference Peak and Common Peak

**Figure 3:**
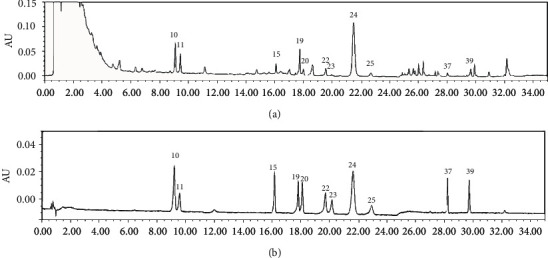
UPLC chromatogram of SMI and mixed reference at 210 nm: (a) samples of SMI (S1); (b) mixed reference (10, ginsenoside Rg1; 11, ginsenoside Re; 15, ginsenoside Rf; 19, ginsenoside Rb1; 20, ginsenoside Rg2; 22, ginsenoside Rb2; 23, ginsenoside Rb3; 24, schisandrol A; 25, ginsenoside Rd; 37, Ophiopogon D; and 39, schisandrin A).

The peak of schisantherin A was used as the reference peak; it showed as an intense peak with preferable chromatographic peak resolution and RRT. Peaks that existed in all SMI samples were appointed as “common peaks.” 44 common peaks were detected in SMI samples, in which 11 peaks were identified (Figure 3): ginsenoside Rg1 (peak #10), ginsenoside Re (peak #11), ginsenoside Rf (peak #15), ginsenoside Rb1(peak #19), ginsenoside Rb2 (peak #20), ginsenoside Rb2 (peak #22), ginsenoside Rb3 (peak #23), schisandrol A (peak #24), ginsenoside Rd (peak #25), Ophiopogonin D (peak #37), and schisantherin A (peak #39).

### 3.3. Similarity of Fingerprints of 9 Batches of SMI

The similarities of all chromatographic patterns among the samples (Table 2) were calculated using software “Chromatographic Fingerprints of Traditional Chinese Medicine, version: 2004A.” The similarities of 9 SMI batches were greater than 0.91. Therefore, our method was precise, stable, reproducible, and reliable.

### 3.4. Putative Targets of SMI Ingredients

**Figure 4:**
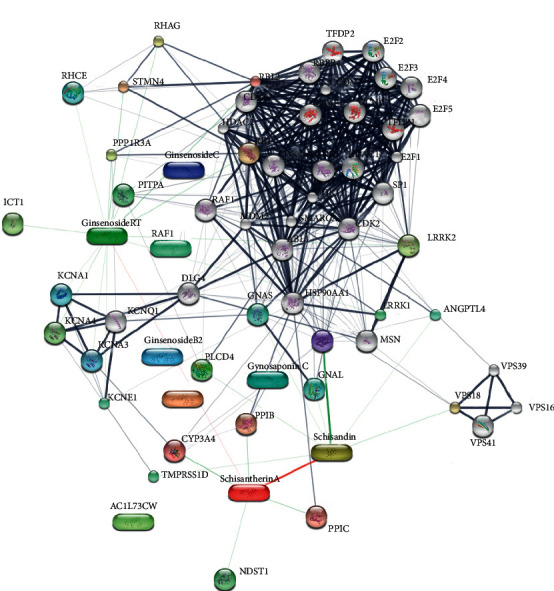
Prediction of component-target of Shengmai injection by STITCH.

The SMI components were screened by TCMSP, and the criteria are OB (oral bioavailability) ≥ 30% and, meanwhile, DL (drug-like) ≥ 0.18 [17–19]. All the components were confirmed in the PubChem database [19] (total 9 bioactive SMI components: ginsenoside Rb1, ginsenoside Rb2, ginsenoside Re, ginsenoside Rf, ginsenoside Rg1, ginsenoside Rg2, schisandrol A, schisantherin A, and Ophiopogon D. Their chemical structures and molecular properties were analyzed and uploaded to the STITCH database for predicting targets that interact with SMI ingredients [20]. A total of 62 targets (Figure 4 and Table 3) showed potential interaction with 9 SMI ingredients.

### 3.5. Acquisition of Known Therapeutic Sepsis Targets

Sepsis targets were collected from the HPO database. The keyword “sepsis” was used to search known therapeutic targets for sepsis in humans [21]. A total of 58 sepsis targets (Table 4) were acquired from the HPO database, and targets were further verified in the NCBI database.

### 3.6. Results of Network Construction

**Figure 5:**
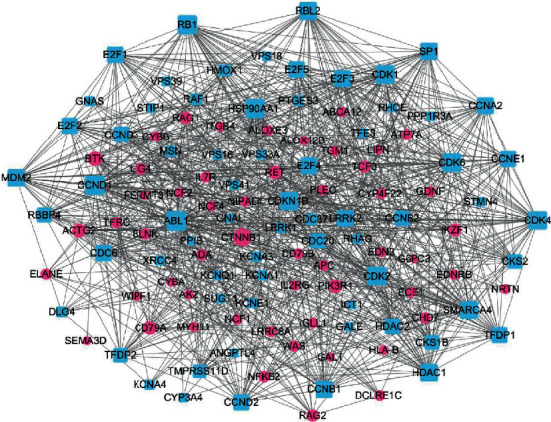
Prediction of protein interaction between SMI and sepsis by STRING (blue-square nodes are targets of SMI; red circular nodes are targets of sepsis).

**Figure 6:**
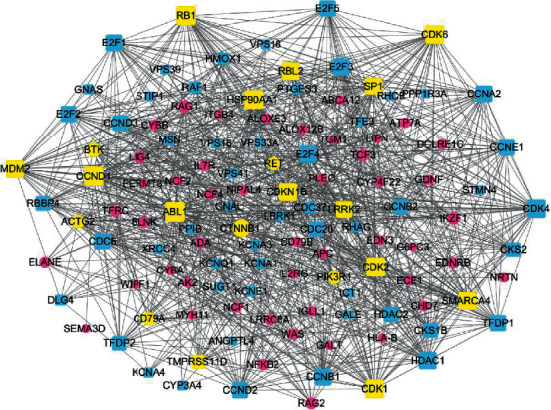
Key targets of the SMI-sepsis protein interaction network (yellow is the key target; blue-square nodes are SMI targets; and red circular nodes are sepsis targets).

The putative targets of SMI active ingredients and sepsis disease targets were determined based on the protein-protein interactions [22]. The interplays amongst SMI targets, known therapeutic targets for sepsis, and interactional human targets were combined to construct the network. The network illustrates the relationship between SMI targets and sepsis targets. The overall interaction network (Figure 5) was visualized using Cytoscape (sepsis targets in red circles and SMI targets in blue squares); the larger a node, the more targets it contains and more important in sepsis management. Targets with higher values of “degree,” “betweenness,” “closeness,” and “coreness” (above the median value of all the network nodes) were identified [23, 24]. Targets which might play unimportant roles in the network according to the topological features were discarded [25]. Median value of “degree,” “betweenness,” and “closeness” was 19, 0.014, and 0.449, respectively. Top twenty proteins were selected as key sepsis therapeutic targets (Figure 6 and Table 5), including ABL1, CCND1, CDK family (CDK1, CDK2, CDK6, and CDKN1B), RB1, HSP90AA1, SMARCA4, RBL2, CTNNB1, MDM2, SP1, LRRK1, BTK, PIK3R1, TMPRSS11D, ACTG2, CD79A, and RET.

Sepsis causes life-threatening organ dysfunction due to a host's complex systemic inflammatory response to infection [26]. In the present study, we identified core proteins that may play important roles in SMI-supportive treatment in sepsis. ABL1 is a tyrosine-protein kinase which is important for cell growth and survival, cytoskeleton remodeling in response to extracellular stimuli, autophagy, and apoptosis [27–29]. It also regulates multiple pathological signaling cascades during infection that alter vascular permeability and the endothelium barrier in inflammation [30].

Cyclin-dependent kinase 1 (CDK1) is a member of the Ser/Thr protein kinase family. Its kinase activity is controlled by cyclin [31, 32]. CCND1/CDK4 and CDK2 are critical for G1/S phase transition. It has been shown rat kidney injury is associated with G1 phase arrest in cecal ligation and puncture- (CLP-) induced sepsis, while upregulation of CCND1/CDK4 and CCNE/CDK2 activates Rb leading to revival of cell cycle progress and recovery of kidney function 48 hours after CLP [33, 34]. The findings demonstrate that cell cycle arrest occurs in sepsis, and drugs that regulate cell cycle proteins may be a means to rescue organ injury [35]. In addition, the targets of SMI are more involved in DNA replication and transcription; for example, MDM2, E3 ubiquitin ligase, mediates ubiquitination and degradation of p53. It mediates apoptosis in organ injury and malignant transformation [36]. SMI may inhibit MDM2 and keep p53 active; therefore, it promotes cells staying in the G1/G2 phase and alleviates cell injury in sepsis.

Molecular chaperone heat-shock protein (HSP 90) is extensively expressed by cells, and its expression increases upon stimulation [37]. HSPs are associated with multiple organ failure in sepsis [38]. In the vast immune response in sepsis, stressed cells release HSPs that are regarded as “danger signal” to neighboring and immune cells [39]. HSP90-*α* has been shown to interact with about 200 client proteins, including signal proteins in the inflammatory pathway such as NF-*κ*B, Akt, and IKK, to interfere inflammation [40–42]. Moreover, HSP90-*α*, as abundant “chaperone,” is one of the main mediators that activates bacteria lipopolysaccharide. It interacts with proteins in the PI3K/Akt pathway and is essential in promoting the immune response and improving host defense to pathogens. Inhibition of HSP90-*α* prevents severe sepsis-associated acute lung injury; therefore, block HSP90-*α* offers a novel treatment for lung injury in sepsis [43–45].

BTK (tyrosine-protein kinase) is a component of the toll-like receptor (TLR) pathway and plays important roles in innate and adaptive immunity. Key target CD79A is required for efficient differentiation of pro- and pre-B-cells. It cooperates with CD79B and bounds to the B-cell antigen receptor complex (BCR) for initiation of the signal transduction cascade. It is pivotal in regulating immunity and inflammation [46]. Network pharmacology revealed SMI represses BTK expression/activation, blocks signals through multiple pathways (TLR, B-cell antigen receptor signal, and apoptosis), and consequently ameliorates cell apoptosis and organ injury. SMI acts as a whole, and each formula has its corresponding targets/syndromes; thus, SMI prescription acts on multiple key targets, and the network pharmacology study of SMI provides insights into understanding its fundamental mechanisms.

### 3.7. KEGG and GO Analysis

To cluster the biological functions of SMI and its targets, data were uploaded to KEGG, and results revealed SMI active formulae target pathways including cancer, cell cycle, p53, B-cell receptor, and ErbB pathways (Table 6). SMI regulates the interplay and synergy among the pathways of immunity, inflammation, and apoptosis to protect cellular and organ injury in sepsis. p53 pathway regulates mitochondrial fission and mitochondrial biogenesis via AMPK, and it alters PKM2-dependent glycolysis. Global deletion of PKM2 results in systemic inflammation in mice [47]. Our GO analysis (Table 7) showed that SMI ingredients regulate multiple biological processes including cell cycle, energy metabolism, cellular signal transduction, transcription regulation, and immunity development. Our data indicate the putative role of SMI in alleviating systemic inflammation and deregulating immunity in the host; moreover, it regulates energy utilization and promotes energy homeostasis and therefore ameliorates multiple organ failure associated with sepsis. It is also in agreement with the idea of SMI used in the early phase of sepsis.

## 4. Conclusion

An UPLC method was developed for analysis of SMI fingerprints. Forty-four peaks were selected as common peaks, of which 11 peaks were identified. The consistency in the chromatograms of 9-batch samples reflects the presence of similar chemical constituents (similarities greater than 0.91). The technique was proven to be useful in SMI quality control. A total of 9 active components of SMI target 20 key proteins including ABL1, CDK, HSP90, BTK, PIK3R1, and CD79A. These proteins are enriched in cell cycle, p53 signaling pathway, B-cell receptor signaling pathway, and ErbB pathway. It is likely that the pharmacological mechanisms of SMI in sepsis treatment are of multiple dimensions that are associated with regulation of cell cycle, energy metabolism, cellular signal transduction, transcription regulation, and immunity development. Further experiments are needed to validate our prediction.

## Figures and Tables

**Table 1:** Mobile phase and proportion of qualitative and quantitative chromatographic conditions.

Time (min)	*A* (%)	*B* (%)
0	19	81
3	19	81
12	26.8	73.2
15	32	68
23	32	68
23.1	44	56
35	66.8	33.2

**Table 2:** Comparison of similarity of Shengmai injection in different batches.

			S1	S2	S3	S4	S5	S6	S7	S8	S9	Control fingerprints
S1			1	0.954	0.983	0.976	0.916	0.938	0.944	0.942	0.953	0.968
S2			0.954	1	0.943	0.945	0.821	0.869	0.89	0.885	0.922	0.914
S3			0.983	0.943	1	0.992	0.931	0.962	0.967	0.965	0.973	0.986
S4			0.976	0.945	0.992	1	0.927	0.945	0.952	0.95	0.967	0.978
S5			0.916	0.821	0.931	0.927	1	0.944	0.944	0.939	0.931	0.963
S6			0.938	0.869	0.962	0.945	0.944	1	0.996	0.996	0.983	0.98
S7			0.944	0.89	0.967	0.952	0.944	0.996	1	0.998	0.989	0.984
S8			0.942	0.885	0.965	0.95	0.939	0.996	0.998	1	0.989	0.98
S9			0.953	0.922	0.973	0.967	0.931	0.983	0.989	0.989	1	0.98
Control fingerprints			0.968	0.914	0.986	0.978	0.963	0.98	0.984	0.98	0.98	1

**Table 3:** Prediction of pharmacodynamic targets of Shengmai injection.

No.	Targets
1	E2F5
2	HDAC2
3	VPS33A
4	TFDP2
5	MSN
6	KCNE1
7	RB1
8	CDK1
9	TFDP1
10	MDM2
11	VPS16
12	VPS39
13	E2F4
14	E2F2
15	CDK2
16	VPS41
17	KCNA4
18	HSP90AA1
19	CDK4
20	CDK6
21	HDAC1
22	CCNA2
23	E2F1
24	RBBP4
25	ABL1
26	SP1
27	CCND2
28	E2F3
29	RBL2
30	CCNE1
31	KCNA3
32	KCNA1
33	RAF1
34	GNAS
35	LRRK1
36	STMN4
37	HMOX1
38	PPP1R3A
39	RHAG
40	PTGES3
41	GNAL
42	CCND1
43	CYP3A4
44	PPIB
45	LRRK2
46	GALE
47	PPIC
48	RHCE
49	VPS18
50	ANGPTL4
51	ICT1
52	DLG4
53	CKS2
54	TMPRSS11D
55	KCNQ1
56	CCNB1
57	CCNB2
58	CCND3
59	CDC20
60	CDC37
61	CDC6
62	CDKN1B

**Table 4:** Prediction of sepsis closely related proteins in the human body.

No.	Targets
1	CYBB
2	RMRP
3	RAG1
4	RAG2
5	TGM1
6	LIG4
7	SEMA3D
8	NIPAL4
9	SEMA3C
10	TCF3
11	MYH11
12	ATP7A
13	WAS
14	WIPF1
15	GALT
16	HLA-B
17	G6PC3
18	DCLRE1C
19	NRTN
20	ABCA12
21	PIK3R1
22	IGHM
23	NFKB2
24	BTK
25	NCF1
26	BLNK
27	APC
28	LRRC8A
29	ELANE
30	ACTG2
31	CYP4F22
32	AK2
33	CD79A
34	CD79B
35	NCF2
36	IKZF1
37	ALOXE3
38	NCF4
39	SERAC1
40	LIPN
41	CHD7
42	IGLL1
43	RET
44	PLEC
45	CTNNB1
46	ECE1
47	ADA
48	IL2RG
49	ITGB4
50	GDNF
51	MUT
52	ALOX12B
53	EDN3
54	EDNRB
55	IL7R
56	FERMT3
57	TFRC
58	CYBA

**Table 5:** Key targets and topological parameters of the network on SMI in the treatment of sepsis.

No.	Gene name	Protein name	Degree	Closeness centrality	Betweenness centrality
1	ABL1	Tyrosine-protein kinase ABL1	43	0.548	0.101
2	CCND1	G1/S-specific cyclin-D1	42	0.522	0.046
3	RB1	Retinoblastoma-associated protein	41	0.509	0.037
4	CDK2	Cyclin-dependent kinase 2	41	0.52	0.026
5	HSP90AA1	Heat-shock protein HSP 90-alpha	40	0.531	0.077
6	CDK1	Cyclin-dependent kinase 1	39	0.509	0.023
7	CDK6	Cyclin-dependent kinase 6	39	0.502	0.015
8	SMARCA4	Transcription activator BRG1	37	0.511	0.037
9	CDKN1B	Cyclin-dependent kinase inhibitor 1B	37	0.506	0.031
10	RBL2	Retinoblastoma-like protein 2	36	0.482	0.04
11	CTNNB1	Catenin beta-1	36	0.509	0.036
12	MDM2	E3 ubiquitin-protein ligase Mdm2	35	0.488	0.025
13	SP1	Transcription factor Sp1	34	0.504	0.027
14	LRRK1	Leucine-rich repeat serine/threonine-protein kinase 1	31	0.515	0.115
15	BTK	Tyrosine-protein kinase BTK	23	0.486	0.038
16	PIK3R1	Phosphatidylinositol 3-kinase regulatory subunit alpha	21	0.47	0.054
17	TMPRSS11D	Transmembrane protease serine 11D	20	0.463	0.041
18	ACTG2	Actin, gamma-enteric smooth muscle	20	0.454	0.025
19	CD79A	B-cell antigen receptor complex-associated protein alpha chain	19	0.458	0.058
20	RET	Proto-oncogene tyrosine-protein kinase receptor Ret	19	0.463	0.032

**Table 6:** Analysis of major metabolic pathways related to sepsis in KEGG.

No.	Name	Count	*p* value
1	Pathways in cancer	11	6.2 × 10^−9^
2	Cell cycle	9	1.2 × 10^−9^
3	p53 signaling pathway	5	4.7 × 10^−5^
4	Melanoma	5	5.6 × 10^−5^
5	Non-small cell lung cancer	4	5.8 × 10^−4^
6	B-cell receptor signaling pathway	3	2.3 × 10^−2^
7	Colorectal cancer	3	2.8 × 10^−2^
8	ErbB signaling pathway	3	3 × 10^−2^

**Table 7:** GO biological analysis.

No.	Name	Count	*p* value
1	Cell cycle	10	3.5 × 10^−7^
2	Phosphorylation	9	6.1 × 10^−6^
3	Phosphorus metabolic process	9	2.6 × 10^−5^
4	Intracellular signaling cascade	9	1.6 × 10^−4^
5	Regulation of transcription	9	1.8 × 10^−2^
6	Mitotic cell cycle	8	4.1 × 10^−7^
7	Protein amino acid phosphorylation	8	2.1 × 10^−5^
8	Positive regluation of macromolecule metabolic process	8	1.0 × 10^−4^
9	Interphase	7	5.0 × 10^−9^
10	Regulation of cell proliferation	7	5.4 × 10^−4^
11	Regulation of transcription, DNA-dependent	7	3.0 × 10^−2^
12	Regulation of RNA metabolic process	7	3.3 × 10^−2^
13	Regulation of mitotic cell cycle	6	2.6 × 10^−15^
14	Hemopoiesis	6	1.5 × 10^−5^
15	Hemopoietic or lyphoid organ development	6	2.4 × 10^−5^
16	Immune system development	6	3.1 × 10^−5^
17	Positive regluation of nitrogen compound metabolic process	6	1.6 × 10^−3^
18	Positive regluation of celluar biosynthetic process	6	2.1 × 10^−3^
19	Positive regluation of biosynthetic process	6	2.2 × 10^−3^
20	Regulation of transcription from RNA polymerase II promoter	6	2.7 × 10^−3^

## Data Availability

The data used to support the findings of this study are available from the corresponding author upon request.

## References

[1] Dardalas I., Stamoula E., Rigopoulos P. (2019). Dexmedetomidine effects in different experimental sepsis in vivo models. *European Journal of Pharmacology*.

[2] Chen X., Feng Y., Shen X. (2018). Anti-sepsis protection of Xuebijing injection is mediated by differential regulation of pro- and anti-inflammatory Th17 and T regulatory cells in a murine model of polymicrobial sepsis. *Journal of Ethnopharmacology*.

[3] Shi H., Hong Y., Qian J., Cai X., Chen S. (2017). Xuebijing in the treatment of patients with sepsis. *The American Journal of Emergency Medicine*.

[4] Li C., Wang P., Zhang L. (2018). Efficacy and safety of Xuebijing injection (a Chinese patent) for sepsis: a meta-analysis of randomized controlled trials. *Journal of Ethnopharmacology*.

[5] Wu W., Jiang R.-l., Wang L.-c. (2015). Effect of Shenfu injection on intestinal mucosal barrier in a rat model of sepsis. *The American Journal of Emergency Medicine*.

[6] Zhang Y.-z., Wu H.-y., Ren L.-w., Zhang H.-s., Jia X., Zhang Y.-z. (2010). Study on modified ShengMai Yin injection for prevention and treatment of brain impairment in endotoxin shock rats. *Journal of Traditional Chinese Medicine*.

[7] Gao Z. L., Yu X. Q., Yang M. (2012). Clinical trial of treating stress-induced hyperglycemia patients with sepsis by supplementing Qi, nourishing Yin, and promoting blood flow. *Chinese Journal of Integrated Traditional and Western Medicine*.

[8] Yu Y. H., Cui N. Q., Wang G. L. (2006). Monocyte response and regulatory effect of emodin and shenmai injection on it in patients with severe sepsis. *Chinese Journal of Integrated Traditional and Western Medicine*.

[9] Yu Y. H., Cui N. Q., Fu Q., Li J. (2005). Change of Th1/Th2 cytokine equilibrium in rats with severe sepsis and therapeutic effect of recombinant interleukin-12 and Shenmai injection. *Chinese Journal of Integrative Medicine*.

[10] Zhan S., Ding B., Ruan Y.-e. (2018). A simple blood microdialysis in freely-moving rats for pharmacokinetic-pharmacodynamic modeling study of ShengMai injection with simultaneous determination of drug concentrations and efficacy levels in dialysate. *Journal of Pharmaceutical and Biomedical Analysis*.

[11] Duan B., Xie J., Rui Q., Zhang W., Xi Z. (2018). Effects of Shengmai injection add-on therapy to chemotherapy in patients with non-small cell lung cancer: a meta-analysis. *Supportive Care in Cancer*.

[12] Yu G. H., Wang W. B., Wang X. (2018). Network pharmacology-based strategy to investigate pharmacological mechanisms of Zuojinwan for treatment of gastritis. *BMC Complementary and Alternative Medicine*.

[13] Cui Y. M., Li C. H., Zeng C. (2018). Tongmai Yangxin pills anti-oxidative stress alleviates cisplatin-induced cardiotoxicity: network pharmacology analysis and experimental evidence. *Biomedicine & Pharmacotherapy*.

[14] Ming H., Sha L., Ning W., Tan H. Y., Cheung F., Feng Y. B. (2017). A biomedical investigation of the hepatoprotective effect of Radix salviae miltiorrhizae and network pharmacology-based prediction of the active compounds and molecular targets. *International Journal of Molecular Sciences*.

[15] Dong Y., Duan L., Chen H. W., Liu Y. M., Zhang Y., Wang J. (2019). Network pharmacology-based prediction and verification of the targets and mechanism for panax notoginseng saponins against coronary heart disease. *Evidence-Based Complementary and Alternative Medicine*.

[16] Tang F., Tang Q., Tian Y., Fan Q., Huang Y., Tan X. (2015). Network pharmacology-based prediction of the active ingredients and potential targets of Mahuang Fuzi Xixin decoction for application to allergic rhinitis. *Journal of Ethnopharmacology*.

[17] Lipinski C. A., Lombardo F., Dominy B. W., Feeney P. J. (2001). Experimental and computational approaches to estimate solubility and permeability in drug discovery and development settings. *Advanced Drug Delivery Reviews*.

[18] Shen M. Y., Tian S., Li Y. Y. (2012). Drug-likeness analysis of traditional Chinese medicines: 1. property distributions of drug-like compounds, non-drug-like compounds and natural compounds from traditional Chinese medicines. *Journal of Cheminformatics*.

[19] Tao W., Xu X., Wang X. (2013). Network pharmacology-based prediction of the active ingredients and potential targets of Chinese herbal radix curcumae formula for application to cardiovascular disease. *Journal of Ethnopharmacology*.

[20] Wu X.-M., Wu C.-F. (2015). Network pharmacology: a new approach to unveiling Traditional Chinese Medicine. *Chinese Journal of Natural Medicines*.

[21] Ma S., Feng C., Zhang X. (2013). The multi-target capabilities of the compounds in a TCM used to treat sepsis and their in silico pharmacology. *Complementary Therapies in Medicine*.

[22] Zhang X. Z., Xiao W., Xu X. J., Wang Z. Z., Cao L., Sun L. (2013). Study on mechanism of the reduning injection on the influenza virus using network pharmacology method. *Acta Physico-Chimica Sinica*.

[23] Zhang Y., Mao X., Su J. (2017). A network pharmacology-based strategy deciphers the underlying molecular mechanisms of Qixuehe capsule in the treatment of menstrual disorders. *Chinese Medicine*.

[24] Li H. Y., Zhao L. H., Zhang B. (2014). A network pharmacology approach to determine active compounds and action mechanisms of Ge-Gen-Qin-Lian decoction for treatment of type 2 diabetes. *Evidence-Based Complementary and Alternative Medicine*.

[25] Zhang A. H. (2014). Identifying potential therapeutic targets of a natural product Jujuboside B for insomnia through network pharmacology. *Plant Science Today*.

[26] Rhodes A., Evans L. E., Alhazzani W. (2017). Surviving sepsis campaign: international guidelines for management of sepsis and septic shock: 2016. *Intensive Care Medicine*.

[27] Valent P., Hadzijusufovic E., Schernthaner G. H., Wolf D., Rea D., Philipp le C. (2014). Vascular safety issues in CML patients treated with BCR/ABL1 kinase inhibitors. *Blood*.

[28] Quintás-Cardama A., Cortes J. (2009). Molecular biology of bcr-abl1-positive chronic myeloid leukemia. *Blood*.

[29] Li Y., Wang H., Tao K. (2013). MiR-29b suppresses CML cell proliferation and induces apoptosis via regulation of BCR/ABL1 protein. *Experimental Cell Research*.

[30] Rizzo A. N., Aman J., van Nieuw Amerongen G. P., Dudek S. M. (2015). Targeting abl kinases to regulate vascular leak during sepsis and acute respiratory distress syndrome. *Arteriosclerosis, Thrombosis, and Vascular Biology*.

[31] Asghar U., Witkiewicz A. K., Turner N. C., Knudsen E. S. (2003). Targets of the cyclin-dependent kinase Cdk1. *Nature*.

[32] Rachael A. M., Samuel R., Elizabeth C., Thierry L., Anna C., Andrew B. (2014). Partial inhibition of Cdk1 in G_2_ phase overrides the SAC and decouples mitotic events. *Cell Cycle*.

[33] Yang Q.-h., Liu D.-w., Long Y., Liu H.-z., Chai W.-z., Wang X.-T. (2009). Acute renal failure during sepsis: potential role of cell cycle regulation. *Journal of Infection*.

[34] Jorrit M. E., Richard D. K. (2010). An overview of Cdk1-controlled targets and processes. *Cell Division*.

[35] Masamitsu F., Steven B. G., Derry W. B., Joel H. R. (2003). Essential embryonic roles of the CKI-1 cyclin-dependent kinase inhibitor in cell-cycle exit and morphogenesis in C elegans. *Developmental Biology*.

[36] Honda R., Tanaka H., Yasuda H. (1997). Oncoprotein MDM2 is a ubiquitin ligase E3 for tumor suppressor p53. *FEBS Letters*.

[37] Christine Q., Todd A. S., Susan L. (2002). HSP90 as a capacitor of phenotypic variation. *Nature*.

[38] Michaela-Diana F., Helen D., Maria V., Marianna K., Stavroula I., George B. (2016). Increased extracellular heat shock protein 90*α* in severe sepsis and SIRS associated with multiple organ failure and related to acute inflammatory-metabolic stress response in children. *Medicine*.

[39] Conway-Morris A., Wilson J., Shankar-Hari M. (2017). Immune activation in sepsis. *Critical Care Clinics*.

[40] Hsu H.-Y., Wu H.-L., Tan S.-K. (2007). Geldanamycin interferes with the 90-kDa heat shock protein, affecting lipopolysaccharide-mediated interleukin-1 expression and apoptosis within macrophages. *Molecular Pharmacology*.

[41] Basso A. D., Solit D. B., Chiosis G., Giri B., Tsichlis P., Rosen N. (2002). Akt forms an intracellular complex with heat shock protein 90 (Hsp90) and Cdc37 and is destabilized by inhibitors of Hsp90 function. *Journal of Biological Chemistry*.

[42] Broemer M., Krappmann D., Scheidereit C. (2004). Requirement of Hsp90 activity for I*κ*B kinase (IKK) biosynthesis and for constitutive and inducible IKK and NF-*κ*B activation. *Oncogene*.

[43] Triantafilou K., Triantafilou M., Dedrick R. L. (2001). A CD14-independent LPS receptor cluster. *Nature Immunology*.

[44] Li X. P., Luo R., Jiang R. J. (2013). The role of the Hsp90/Akt pathway in myocardial calpain-induced caspase-3 activation and apoptosis during sepsis. *BMC Cardiovascular Disorders*.

[45] Chatterjee A., Dimitropoulou C., Drakopanayiotakis F. (2007). Heat shock protein 90 inhibitors prolong survival, attenuate inflammation, and reduce lung injury in murine sepsis. *American Journal of Respiratory and Critical Care Medicine*.

[46] Pone E. J., Zhang J. S., Mai T. (2012). BCR-signalling synergizes with TLR-signalling for induction of AID and immunoglobulin class-switching through the non-canonical NF-*κ*B pathway. *Nature Communications*.

[47] Huang J., Liu K., Zhu S. (2018). AMPK regulates immunometabolism in sepsis. *Brain, Behavior, and Immunity*.

